# Medicinal Plants from Brazilian Cerrado: Antioxidant and Anticancer Potential and Protection against Chemotherapy Toxicity

**DOI:** 10.1155/2019/3685264

**Published:** 2019-08-25

**Authors:** José Tarcísio de Giffoni de Carvalho, Débora da Silva Baldivia, Daniel Ferreira Leite, Laura Costa Alves de Araújo, Priscilla Pereira de Toledo Espindola, Katia Avila Antunes, Paola Santos Rocha, Kely de Picoli Souza, Edson Lucas dos Santos

**Affiliations:** Research Group on Biotechnology and Bioprospecting Applied to Metabolism, Federal University of Grande Dourados, Dourados, Brazil

## Abstract

The use of natural antioxidants in cancer therapy has increased: first, due to the potential of natural antioxidants to kill tumour cells and second, because of their capacity to protect healthy cells from the damage caused by chemotherapy. This review article discusses the antioxidant properties of extracts obtained from medicinal plants from the Brazilian Cerrado and the cell death profile induced by each of these extracts in malignant cells. Next, we describe the capacity of other medicinal plants from the Cerrado to protect against chemotherapy-induced cell toxicity. Finally, we focus on recent insights into the cell death profile induced by extracts from Cerrado plants and perspectives for future therapeutic approaches.

## 1. Introduction

Natural products or their derivatives represent approximately 60% of all chemotherapeutic agents approved by the Food and Drug Administration (FDA), including vincristine, vinblastine, and Taxol [1–3]. However, the search for medicinal plants with anticancer properties has intensified in recent years since chemotherapeutic agents are limited by a high rate of drug resistance and by severe side effects. Additionally, some of the current drugs used in cancer therapy are very expensive to produce [2, 4]. Therefore, there is great interest in the discovery and identification of effective anticancer compounds and molecules with low production costs and high target cell selectivity [5–7].

Brazil is considered to be the territory with the richest biodiversity in the world [8–11]; the Cerrado is the second main biome, exhibiting a great diversity of natural plants [9, 12, 13]. The Cerrado is located in the middle west of Brazil, encompassing almost 2 million km^2^ that covers 21% of the Brazilian territory [14, 15].

Numerous studies have evaluated the biological effects of extracts from medicinal plants from the Cerrado. These extracts include *Stryphnodendron adstringens*, popularly known as barbatimão, which has displayed antiulcerogenic and antifungal effects [16], and *Campomanesia adamantium*, popularly known as Guavira, which has presented antidiabetic properties, anti-inflammatory, and diuretic actions [17]. Another plant from the Brazilian Cerrado is *Senna velutina*; little is known about its biological effects, but an important study investigated its antitumour activity in a leukaemia cell lineage [18]. In addition, the *Jacaranda* [19] and *Harconia* [20] genera are other examples of medicinal plants from the Cerrado commonly used in folk medicine and with some described biological properties, mainly antioxidant activity. Finally, *Schinus terebinthifolius* and *Guazuma ulmifolia* are used in traditional medicine to treat ulcers, diarrhoea, arthritis, and infections [21] and inflammation, gastrointestinal diseases, and diabetes [22], respectively. The botanical features and geographical distribution of plants from the Brazilian Cerrado are outlined in Table 1.

This review article discusses the antioxidant properties of extracts obtained from medicinal plants from the Brazilian Cerrado and the cell death profile induced by these extracts in malignant cells. Next, we describe the capacity of other medicinal plants from the Cerrado to protect against chemotherapy-induced cell toxicity.

## 2. Redox Balance Potential

Natural antioxidants are molecules that protect cells from the damage induced by reactive oxidative species (ROS) [4, 66]. These ROS, including superoxide anion (O_2_^•^−) and hydrogen peroxide (H_2_O_2_), are involved in various cellular processes (host immune defence, cell signaling, cellular respiration process, and others); however, if they are not properly regulated by the antioxidant system, ROS initiate a number of deleterious effects, which may cause the oxidation of biomolecules [67, 68]. For example, excessive ROS results in lipid peroxidation, a process in which free radicals attack polyunsaturated fatty acids, a lipid present in the cell membrane, resulting in membrane rupture and the production of toxic molecules, especially malondialdehyde (MDA), associated with cell damage and mutagenicity [68–70].

Superoxide is generated from diverse metabolic pathways in cells, including the mitochondrial respiratory chain and the enzymatic action of cytochrome p450 and NADPH oxidases [71, 72]. The superoxide that results from these reactions can undergo dismutation to generate water (H_2_O) by superoxide dismutase (SOD), an enzyme in the antioxidant system or can react with nitric oxide (NO^•^), to generate reactive nitrogen species, such as peroxynitrite (ONOO^−^), the most powerful oxidant [67, 69].

Superoxide dismutase catalyses the dismutation of O_2_^•−^ to hydrogen peroxide (H_2_O_2_), which is a less reactive species and a substrate for other enzymes involved in the antioxidant system. Successively, in a Fenton reaction, H_2_O_2_ can be modified to a toxic hydroxyl radical (OH^−^) in the presence of transmission metals, such as iron (Fe^2+^), and therefore should be decomposed to H_2_O. For this step, the most efficient enzymatic antioxidants are catalase (CAT) and/or glutathione peroxidase enzymes (GPx) [73, 74]. GPx reduce peroxides to water (or alcohol) through oxidation of selenol residue to selenenic acid (RSe-OH) groups which are converted back to selenols by the tripeptide glutathione (GSH). Oxidazed gluthatione (GSSH) is oxidazed back to GSH by glutatione reductase [67, 69, 73].

Numerous studies have evaluated the antioxidant potential of extracts from plants from the Cerrado. Campos et al. [18] studied the effects of *S. velutina* on radical scavenging activity. Extracts prepared from the leaves of *S. velutina* in an ethanol solvent were found to be very potent inhibitors of radical scavenging activity by the DPPH (2,2′-diphenyl-1-picrylhydrazyl) method, and the concentration necessary for the 50% inhibition (IC_50_) of DPPH of these extracts was lower than that of the commercial antioxidant butylated hydroxytoluene (BHT) (6.3 ± 1.3 versus 21.3 ± 1.2 *μ*g/mL). Similarly, Dos Santos et al. [46] evaluated the antioxidant capacity of a leaf extract of *Hancornia speciosa* in an ethanolic solvent and also observed a potential activity by the DPPH method and improved IC_50_ values in relation to BHT (9.4 ± 0.8 versus 66.1 ± 23.6 *μ*g/mL).

Espindola et al. [38] found that an extract prepared from the root of *C. adamantium* in an aqueous solvent and BHT had similar antioxidant capacities by the DPPH method (IC_50_ : 37.3 ± 4.1 versus 36.1 ± 9.1 *μ*g/mL) [38]. Baldivia et al. [16] evaluated the antioxidant effects of an extract from the stem bark of *S. adstringens* by the DPPH and ABTS methods (2,2′-azino-bis(3-ethylbenzothiazoline-6-sulfonic acid)). The antioxidant efficacy of *S. adstringens* is similar to that of ascorbic acid according to both methods (DPPH IC_50_, 3.81 ± 0.02 versus 2.65 ± 0.03 *μ*g/mL; ABTS IC_50_, 1.83 ± 0.15 versus 1.34 ± 0.01 *μ*g/mL).

These results demonstrate that the extracts obtained from *S. velutina*, *H. speciosa*, *C. adamantium*, and *S. adstringens* may directly react with free radicals by electron donation radical scavenging, thereby inhibiting ROS-induced damage. These actions can be attributed to the presence of phenolic compounds. The antioxidant efficiency of a phenolic compound depends on the capacity of a hydrogen atom in a hydroxyl group on an aromatic structure to be donated to a free radical [75, 76]. Among the phenolic compounds described as major potential antioxidants, gallic acid is a well-described phenolic compound with antioxidant and antihaemolytic activities in human erythrocytes [77–79]. Procyanidins are also excellent antioxidants capable of protecting erythrocytes from oxidative haemolysis [80, 81]. Furthermore, flavonoids known as catechins [82, 83], rutin [84, 85], and quercetin [86, 87] are among the most abundant and important chemical constituents of plant species and are described as lipid peroxidation inhibitors. The phenolic compounds identified in extracts of *C. adamantium*, *S. velutina*, and *S. adstringens* are listed in Table 2.

The extracts from these plants also showed antioxidant activity and demonstrated lipid peroxidation prevention in 2,2′-azobis(-amidinopropane) dihydrochloride- (AAPH-) induced erythrocyte haemolysis as evidenced by MDA production. Importantly, MDA is related to cell damage and mutagenicity and the inhibition of this process can restore cell homeostasis and prevent the development of oxidative stress-related disease [88].

Casagrande et al. [19] evaluated the activities of SOD, CAT, and GPx antioxidant enzymes in human erythrocyte lysates and found that a hydroethanolic extract of *Jacaranda decurrens subsp. symmetrifoliolata* leaves increased the enzyme activity of glutathione peroxidase and reduced the activity of superoxide dismutase and catalase. Rocha et al. [21] showed that the enzymatic activity of SOD and GPx enzymes increased upon treatment of human erythrocytes with an extract prepared from the leaves of *S. terebinthifolius* in methanol solvent. Based on these results, it seems that *J. decurrens* and *S. terebinthifolius* may modulate the endogenous antioxidant system.

Extracts of *C. adamantium*, in addition to scavenging activity, were described to reduce MDA *in vitro* and *in vivo* [38]. The capacity of *C. adamantium* to present *in vivo* antioxidant effects represents a major advantage for the development of new products. In many cases, *in vitro* findings are not reproduced in an organism due to various factors, such as enzyme inactivation, poor absorption, and tissue distribution [89, 90]. This finding suggests that the *C. adamantium* extract showed a bioavailability profile suitable for use *in vivo*.

These results demonstrated that these extracts are very potent antioxidants due to their radical scavenging capacity and their capacity to protect the cell against lipid peroxidation. Furthermore, the synthetic antioxidants commonly used are reported to be mutagenic and cause liver injury [91]. The search for new antioxidants that are more effective and have a better toxicity profile than current antioxidants is desirable, and the plants described here may represent interesting targets for this purpose.

## 3. Antioxidants and Cancer

Cancer is a multistage process resulting in an uncontrolled cell cycle and cell division and apoptosis resistance and is one of the main diseases that cause mortality worldwide [92]. Carcinogenesis is a process that involves multiple steps, including an initiation phase that can occur after exposure to a carcinogenic agent, and commonly results in increased production of ROS [93, 94]. The initialization of cancer cells commonly depends on mutations in genes related to the regulation of the cell cycle, apoptosis, and/or growth factor signalling pathways, which can be induced by ROS-mediated DNA mutations [95, 96].

The interaction between antioxidants and cancer cells can occur in at least three ways:

- Prevention: the ability of antioxidants to protect cells from ROS-induced DNA damage is the basis of the association of antioxidants with cancer prevention [97–99]
- Protects against chemotherapy toxicity: chemotherapy commonly increases the production of ROS, which induces oxidative stress in cancer cells and other tissues. Excessive ROS may cause a disruption in cellular homeostasis, which can lead to toxicity. Therefore, to improve the clinical response to chemotherapy, combination approaches with antioxidants are being investigated by providing protection against toxic side effects [100, 101]
- New anticancer molecules: recent evidence has suggested that antioxidants can also be used to eliminate cancer cells. Over the last few decades, antioxidant extracts from medicinal plants have shown a great cytotoxic potential [102, 103]

## 4. Cell Death Pathway in Cancer Cells

Currently, cell death continues to be considered a complex process that results in a variety of pathways [104–106]. The fact that cells die through different death pathways and that cancer cells can be resistant to each cell death signalling pathway is a relevant aspect in the development of new drugs for anticancer therapy [107, 108]. To date, knowledge of the cell death pathway induced by medicinal plants from the Cerrado is still scarce [109, 110].

### 4.1. Classical Cell Death

Apoptosis is a regulated and controlled process accompanied by a series of hallmarks, including cell shrinkage, chromatin condensation, DNA fragmentation, and apoptotic body formation, which is dependent on the activation of a protease enzyme family called caspases [111, 112]. In apoptosis, a change in the membrane of the cell marks the cell for recognition and phagocytosis by macrophages [113, 114].

Despite the modulation of apoptosis by drugs in cancer cells, the activation of the intrinsic pathway is a critical step [109, 115]. In response to insults, the opening of pores occurs in the mitochondrial membrane and the release of proapoptotic factors, such as cytochrome c, then forms an apoptosome complex in the cytosol together with the apoptosis inductor factor and pro-caspase-9, leading to caspase-9 activation. Caspase-9 then activates effector caspases such as caspase-3, resulting in the cleavage of several cellular targets involved in all aspects of apoptosis. The release of proapoptotic factors from mitochondria is regulated by proapoptotic (BAX) and antiapoptotic (Bcl-2) proteins [116–118].

In addition to the Bcl-2 family, the intrinsic pathway can also be modulated by intracellular calcium [119–121] and the ROS generated by mitochondria [71, 72, 122]. The ROS generated by mitochondria, or elsewhere in the cells, can activate p53, which activates proapoptotic Bcl-2 proteins that can inhibit the functions of antiapoptotic proteins [71, 122–124]. Moreover, ROS cause mitochondrial membrane depolarization and/or open Bax/Bak channels on the mitochondrial membrane, which allows for the release of apoptosis-inducing factor, endonuclease G, cytochrome c, and Smac/Diablo into the cytosol [72, 124]. Furthermore, the perturbation of intracellular Ca^2+^ homeostasis is also associated with cell death. Endoplasmic reticulum stress responses can induce lesions that affect membrane integrity and the release of Ca^2+^ [120, 121, 125]. Following Ca^2+^ efflux into the cytoplasm, the proapoptotic proteins Bak and Bax, which are located in both the reticulum and mitochondria, may be delivered to the cytosol. Calcium overload can induce mitochondrial dysfunction and cell death accompanied by membrane rupture, a process called necrosis [119, 125].

### 4.2. Alternative Cell Death Pathway

For several decades, apoptosis was depicted as programmed cell death in malignant and healthy cells and as a pivotal target for new therapies. Recently, other forms of cell death have also been increasingly noted [111, 126]. Discovering novel therapeutic strategies that may induce alternative cell death pathways appears to be especially useful for opposing malignant cell resistance to caspase-dependent apoptosis [127].

Necroptosis is a form of necrosis that occurs under caspase-deficient conditions [128, 129]. At the molecular level, necroptosis depends on the activation of serine/threonine receptor-interacting protein kinases 1 and 3 (RIPK1 and RIPK3) by death receptor ligands, which leads to the activation of mixed lineage kinase domain-like pseudokinase (MLKL) [130–132], allowing for a cascade of intracellular events involving Ca^2+^ influx, ROS production, and membrane rupture [133]. Moreover, accumulating evidence has shown that necroptosis promotes an anticancer immune response [134].

Lysosome-dependent cell death is initiated by perturbations of intracellular homeostasis and is demarcated by the permeabilization of lysosomal membranes [135–137]. Upon lysosomal stress, lysosome-dependent cell death proceeds through membrane permeabilization, resulting in the release of proteolytic enzymes from the cathepsin family to the cytoplasm, which activates death signalling pathways. More commonly, ROS play a prominent causal role in lysosomal permeabilization. The production of hydroxyl radicals by Fenton reactions destabilizes the lysosomal membrane upon lipid peroxidation, but an increase in cytoplasmic Ca^2+^ is also a key regulator reportedly involved in the activation of lysosomal cell death [138, 139]. Moreover, lysosomal dysregulation may be associated with alterations in autophagy and the role of ROS in homeostasis and cell death [137]. Autophagy is a self-digestive process that involves lysosomal fusion to degrade unnecessary or dysfunctional cellular components [140]. The role of autophagy in cancer is controversial; thus, the modulation of autophagy depends on each subtype of malignant cells and an improved understanding of this pathway in the cancer environment [141].

## 5. Cell Death Profile Induced by Plants from the Cerrado

**Figure 1:**
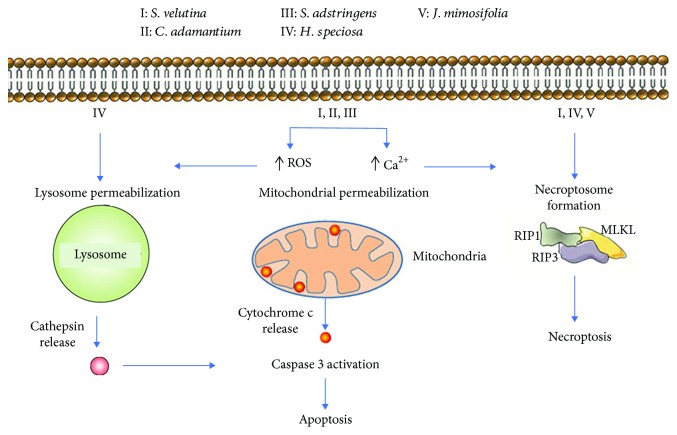
Cell death profile induced by extracts and/or compounds from medicinal plants of Cerrado.

Several reports from our group [16–19] have demonstrated the potential anticancer properties of medicinal Cerrado plants. Assessing the cell death profile induced by these extracts, through cell death inhibitors and/or caspase detection indicated the involvement of different cell death pathways for each plant extract. For example, many studies have demonstrated the antitumour potential of plant extracts through caspase-independent cell death, including *H. speciosa* [20] and *J. decurrens* [19], while others such as *C. adamantium* [17], *S. velutina* [18], and *S. adstringens* [16] killed malignant haematologic cells or melanoma cells through apoptosis (Figure 1 and Table 2).

Campos et al. [18] studied the effect of an extract from the leaves of *S. velutina* in two leukaemia cell lines: Jurkat cells, acute T cell leukaemia cells, and K562, Philadelphia chromosome-positive cells. Jurkat cells were found to be more sensitive to the cytotoxic effect of *S. velutina* than K562 cells, and this effect was accompanied by caspase-3 activation, mitochondrial depolarization, and cell cycle arrest at the S and G2 phases. Furthermore, these features were reversed by chelation of calcium, demonstrating the involvement of calcium as the main regulator of cell death mediated by *S. velutina*. Castro et al. [142] evaluated the effect of an extract from the roots of *S. velutina* on a melanoma cell line B16F10-Nex2 and also evaluated the antimetastatic effect of this extract using models of tumour volume progression and pulmonary nodule formation in C57Bl/6 mice. The extract reduced cell viability and promoted apoptotic cell death, caspase-3 activation, with increased intracellular calcium and ROS levels, and cell cycle arrest at the sub-G0/G1 phase. *In vivo*, the tumour volume progression and pulmonary metastasis of *S. velutina*-treated mice decreased by over 50%. Taken together, these results show that *S. velutina* had *in vitro* and *in vivo* antitumour effects, predominantly through apoptosis, thus demonstrating its promising potential as a therapeutic agent in the treatment of melanoma, leukaemia, and possibly other types of cancer.

Other studies have identified the *in vitro* antiproliferative activity of the extract from leaves of *C. adamantium* in many cell lineages, including murine melanoma cells (B16-F10) [143], prostate cancer cells (PC-3) [144], breast adenocarcinoma cells (MCF-7), cervical adenocarcinoma cells (HeLa), and glioblastoma cells (M059J) [145]. However, none of these studies evaluated the cell death profile induced by extract of *C. adamantium*. In another study, Campos et al. [17] studied the cell death profile induced in Jurkat cells by an extract prepared from the leaves and roots of *C. adamantium*. A dose-dependent inhibition of viability occurred in cells incubated with the leaves and roots of *C. adamantium*. This effect was dependent on the accumulation of cytosolic Ca^2+^ and on cell cycle arrest at the S phase. In addition, the cell death induced by extracts was likely mediated by the intrinsic apoptotic pathway, since both extracts induced the activation of caspase-9 and caspase-3, and cell death was reversed after incubation with a general caspase inhibitor.

Baldivia et al. [16] found a similar profile of cell death in a study evaluating the effect of a hydroethanolic extract of the stem bark from *S. adstringens* on the melanoma cell line B16. *S. adstringens* increased the production of ROS, which may have induced the disruption in mitochondrial membrane potential that caused the apoptotic cell death observed in melanoma cells. These *in vitro* experiments demonstrated that *S. adstringens* is a potent cytotoxic extract that induces apoptosis-mediated cell death [16]. Kaplum et al. [146] investigated the *in vitro* anticancer activity of a proanthocyanidin polymer-rich fraction of the stem bark from *S. adstringens* (extracted in acetone : water) in cervical cancer cell lines, including HeLa (HPV18-positive), SiHa (HPV16-positive), and C33A (HPV-negative) cells, and evaluated *in vivo* anticancer activity. HeLa and SiHa cells treated with the extract exhibited intense oxidative stress, mitochondrial damage, and increased Bax/BCL-2 ratio and caspase-9 and caspase-3 expression. The inhibition of ROS production by N-acetylcysteine significantly suppressed oxidative stress in both cell lines. *In vivo*, the extract significantly reduced tumour volume and weight of Ehrlich solid tumours and significantly increased lipoperoxidation, indicating that it also induced oxidative stress in the *in vivo* model. These findings indicate that the proanthocyanidin polymer-rich fraction of *S. adstringens* may be a potential chemotherapeutic candidate for cancer treatment. Sabino et al. [147] investigated the *in vitro* anticancer activity of a fraction isolated from an aqueous leaf extract of *S. adstringens* in breast cancer cell lines. The fraction was cytotoxic against two human breast cancer cell lines: the estrogen receptor-positive cell line MCF-7 and the triple-negative cell line MDA-MB-435. Treatment with the fraction increased the expression of Bax, caspase-9, active caspase-3, caspase-8, LC-3, and beclin-1 and decreased the expression of Bcl-2, caspase-3, and pro-caspase-8 in cancer cells. Taken together, these results show that *S. adstringens* had *in vitro* and *in vivo* antitumour effects, predominantly through apoptosis, thus demonstrating its promising potential as a therapeutic agent in the treatment of melanoma, cervical cancer, breast cancer, and possibly other types of cancer.

Despite the benefits of the pharmacological cancer therapies, the high toxicity of chemotherapeutic drugs is one of the main identified problems. Importantly, in this context, *S. adstringens* and *C. adamantium* show less toxicity against healthy normal cells and peripheral blood mononuclear cells (PBMCs) than tumour cells. In particular, extracts made from the leaves of *C. adamantium* did not change the viability of PBMCs at the evaluated concentrations but exhibited an IC_50_ of 40 *μ*g/mL in Jurkat cells. Although there was a cytotoxic effect of *S. adstringens* on PBMCs, this effect only occurred at the highest concentration evaluated (≥200 *μ*g/mL), which is comparable to the IC_50_ (65 *μ*g/mL) exhibited against B16 cells, suggesting a high therapeutic index. These experiments indicate that these plants show selective effects against cancer cells and possibly do not confer any toxicity to healthy normal cells. New targeted therapies with low toxicity and limited side effects are promising for the development of new anticancer agents [148].

Dos Santos et al. [46] evaluated the cell death profile of an extract from *H. speciosa* in an acute myeloid leukaemia cell line, Kasumi-1. The extract from *H. speciosa* promoted caspase-independent apoptosis because the pancaspase inhibitor did not inhibit the cytotoxic activity of these extracts. This extract killed Kasumi-1 through the involvement of cathepsins and necroptosis and consequently, an alternative pathway of cell death. Cell signalization dependent on lysosomal degradation remains not yet understood, and it seems to modulate autophagic flux [137]. Thus, additional studies evaluating the modulation of autophagic flux mediated by *H. speciosa* are desirable.

Casagrande et al. [19] evaluated the cell death profile induced by extracts from *J. decurrens* in K562 erythroleukaemia cells. These researchers found concentration-dependent cytotoxic activity against the malignant cells studied, which occurred through late apoptosis and necrosis, the activation of caspase-3, and a decrease in mitochondrial membrane potential. Clinically, this cell death pathway (necrosis and necroptosis) is promising for the development of new anticancer compounds against malignant cells resistant to apoptosis [149, 150]. Moreover, accumulating evidence has shown that necroptosis promotes an anticancer immune response [134]. The great potential of necroptosis induced by *H. speciosa* and *J. decurrens* suggests further evaluation of the immunogenicity capacity of these medicinal plants.

Many phenolic compounds derived from Cerrado plants have demonstrated potential anticancer properties. At high concentrations, the phenolic compounds can act as prooxidants and impair the redox balance of malignant cells [151–155]. Gallic acid is a phenolic compound found in both *C. adamantium* and *S. adstringens*. Gallic acid induces death in various cell lines via the intrinsic apoptotic pathway [79–81]. *S. velutina* also has a large number of phenolic compounds; specifically, the phytochemical analysis of roots identified flavonoid-like molecules, such as epigallocatechin, epicatechin, rutin, kaempferol glycosides, and dimeric and trimeric proanthocyanidins [18], and identified the main compounds to be the flavonoid derivatives of catechin and piceatannol (active metabolite of resveratrol) groups and dimeric tetrahydroanthracene derivatives [142].

Accordingly, several flavonoids, such as luteolin, jacaranone, triterpenes, ursolic acid, and oleanolic acid, have been identified in the genus *Jacaranda* and their cytotoxic activities have been described [155–158]. Further studies to isolate and identify the compounds in the medicinal plants *H. speciosa*, *J. decurrens*, *S. velutina*, *C. adamantium*, and *S. adstringens* and clinical trials to study these extracts and/or isolated compounds have potential to facilitate the development of alternative therapeutic strategies and the design and selection of new drugs for cancer therapy.

## 6. Protection against Chemical Toxicity by Plants from the Cerrado

Chemotherapy commonly increases the production of ROS, which induces oxidative stress in cancer cells and other tissues [160, 161]. Excessive ROS may disrupt cellular homeostasis, leading to toxicity [162–164]. In fact, after chemotherapy treatment, oncology patients exhibit signs of lipid peroxidation in plasma, reduced levels of antioxidant vitamins in the blood, and decreased levels of GSH in tissues [165]. For example, drugs such as taxanes (paclitaxel and docetaxel) and vinca alkaloids (vincristine and vinblastine) induce cell death by cytochrome c release from mitochondria and interfering with the electron transport chain, resulting in the production of superoxide radicals [166]. Other drugs, such as anthracyclines (for example, doxorubicin), also generate extremely high ROS levels [167].

Combinatory approaches with antioxidants can protect the health tissues against toxic side effects, improving the clinical response of chemotherapy [164, 168–170]. Regardless of the role of plant antioxidants from the Cerrado in chemotherapy, two recent studies evaluated the effect of doxorubicin on chemotherapy using *in vitro* and *in vivo* models. Dos Santos et al. [22] evaluated the capacity of *G. ulmifolia* extract to protect against doxorubicin injury *in vitro* and *in vivo*. The oxidative stress markers in human erythrocytes exposed to doxorubicin, including haemolysis and MDA, were reduced by the combined use of *G. ulmifolia* extract and doxorubicin. *G. ulmifolia* extract also induced cardioprotection in rats treated with doxorubicin. *G. ulmifolia* extract was able to prevent MDA production in the cardiac tissue of animals treated with doxorubicin. Similarly, Rocha et al. [21] described the potential of *S. terebinthifolius* to protect against doxorubicin injury *in vitro* and *in vivo*. The treatment of C57Bl/6 mice with a *S. terebinthifolius* leaf extract protected against doxorubicin-induced cardiotoxicity, corroborating the results of the reduced oxidative haemolysis *in vitro*. The cotreatment of doxorubicin with *G. ulmifolia* or *S. terebinthifolius* did not attenuate cytotoxicity in erythroleukaemic cells, confirming that these antioxidants do not specifically interfere with the cytotoxic efficacy of this anticancer agent. In conclusion, *G. ulmifolia* and *S. terebinthifolius* have been found to be capable of protecting against the damage caused by doxorubicin and can offer a therapeutic opportunity for treating cancer.

Other studies have evaluated the antimutagenic potential of some plants from the Cerrado. As discussed previously, carcinogenesis initiation, progression, and promotion are processes related to increased intracellular ROS. Martello et al. [171] described the antimutagenic activities of *C. adamantium* hydroethanolic extract in Swiss mice treated with cyclophosphamide. When the extract was administered in combination with cyclophosphamide, the micronucleus frequency and apoptosis were reduced. Extract components might affect cyclophosphamide metabolism, which possibly leads to the increased clearance of this chemotherapeutic agent. Thus, caution should be exercised when consuming these extracts, especially when received in combination with other drugs. de Oliveira et al. [172] investigated the capacity of *C. adamantium* fruits to protect HepG2 cells (hepatocytes) from carbon tetrachloride- (CCl_4_-) induced toxicity. Carbon tetrachloride (CCl_4_) is a highly toxic chemical that is used to investigate hepatotoxicity. Pretreatment of HepG2 cells with pulp or peel/seed hydroalcoholic extract significantly protected against the cytotoxicity induced by CCl_4_. Additionally, the cells treated with both extracts (both at 1000 *μ*g/mL) showed normal morphology (general and nuclear), in contrast to the apoptotic characteristics of the cells only exposed to CCl_4_ [172].

In another study, using a similar model, Abdou et al. [173] found that the administration of an ethanol extract of leaves from *S. terebinthifolius* significantly protected against CCl_4_ liver damage in Wistar rats. Interestingly, *S. terebinthifolius* extract inhibited hepatocyte apoptosis as revealed by an approximate 20-time downregulation in caspase-3 expression compared with the CCl_4_-untreated group. Endringer et al. [97] investigated the capacity of *H. speciosa* to induce antioxidant response element (ARE) activation in HepG2 cells transfected with ARE-luciferase plasmid. ARE is a regulatory enhancer gene encoding protective proteins, including phase II detoxification enzymes such as NAD(P)H:quinone oxidoreductase and antioxidant enzymes such as glutathione (GSH) S-transferases (GST). Extracts and fractions (methanol and methanol : water (1 : 1)) caused ARE induction.

## 7. Conclusion

The evidence discussed in this review indicates that the medicinal plants from the Cerrado show antioxidant activity, anticancer activity, and protective effects against chemical toxicity. These plants are potential candidates for the identification of effective pharmacological compounds. Therefore, the in vivo assay followed by clinical trials may provide clear evidence on the potential benefits of these extracts and/or isolated compounds and may facilitate the development of alternative therapeutic strategies and the design and selection of new drugs for cancer therapy.

## Figures and Tables

**Table 1:** Biological activities, botanical features, and geographical distribution of plants from Brazilian Cerrado.

Name	Family	Biological activities	Botanical features	Geographical distribution
*Stryphnodendron adstringens* Mart. Coville	Fabaceae	The roots and stem bark extract (h) have wound healing [23, 24], anti-inflammatory (w) [25], antigastric ulcer (e) [26], and antidiabetic (e) [27] effects, and fractions from stem bark extract have antimicrobial activity against *Cryptococcus neoformans* (aw-F/etacF) [28], *Candida albicans* (aw-F/etacF) [29, 30], herpes virus (etacF) [31], and gram-positive bacteria (e, h, and b) [32].	Trees in this genus are medium-sized, the trunk does not have ramifications, and the stem usually has a rusty, coarse, and rust-colored bark. Species can be differentiated by their leaf structure; *S. adstringens* has 5–7 pairs of leaflets in an opposite sense with 5–6 pairs of second-order leaflets alternately inserted, different from other species [33].	Distribution of gender is limited to the area between Nicaragua and the southern regions of Brazil. S. adstringens more specifically is distributed in Brazilian states: Tocantins, Bahia, Distrito Federal, Goiás, Mato Grosso, Minas Gerais, São Paulo, and Paraná [33].

*Campomanesia adamantium* O. Berg	Myrtaceae	The peel extract (e : w–70/30%) was found to have antiplatelet and anti-inflammatory effects [34]; the seed extract (w) has an antinociceptive effect [35, 36]; the peel extract (e : w–70/30%) has antidiarrhoeal activity [37]; and the leaf extract (w) has hypolipidaemic [38] effects.	They are shrubs with elliptical branches, growing from 1.5 m to 3 m in height with a disorganized crown. The trunk is tortuous and branched from the base with yellowish bark. The leaves are simple, opposing, oblong (longer than broad), and glabrescent (with almost no hair on the mature leaf), and the dimensions of the leaf range are from 4.5 to 6.8 cm in length by 1.5 to 2.3 cm wide [39].	Endemic distribution in some Brazilian states such as Minas Gerais, Mato Grosso do Sul, and Santa Catarina and arriving in some adjacent regions in Argentina and Paraguay [39].

*Hancornia speciosa* Gomes	Apocynaceae	The bark extract has antidiabetic, antiobesity, antimicrobial, and gastroprotective activities (he) [40]; the latex has anti-inflammatory activity [41]; and the leaf extract (e) was found to have antihypertensive [42], vasodilatory (e) [43, 44], and antidiabetic (e) [45] activities.	*H. speciosa* is a tree that has a medium-sized (2-10 m), tortuous, and rough trunk. The leaves are simple, alternating, and opposite and varied in shapes and sizes. The flowers are white and have an elongated shape. The small fruit has a shape similar to that of the pear [46].	Widely distributed throughout the Brazilian territory and described in other countries such as Paraguay, Peru, Bolivia, and Venezuela [46].

*Schinus terebinthifolius* Raddi	Anacardiaceae	The leaf extract (e) has antidiabetic activity [47]. The leaf extracts (me) can treat neuropathic pain [48] and have antihypertensive (meF) [49] and antiarthritis (he) [50] effects. The essential oil from twigs and leaf extract (c : m) showed activity against *Enterococcus faecium* and *Streptococcus agalactiae* [51], and the essential oil from leaves (e) has anti-inflammatory and wound healing effects [52]. Isolated endophytic fungi showed activity against *Staphylococcus aureus* and *Pseudomonas aeruginosa* [53]. The stem bark extract has activity against herpes simplex virus type 1 (he and mef) [54]; the bark extract has activity against the Candida genus (a) [55]; and the leaves and fruits extracts (e) have activity against *Escherichia coli* [56].	*S. terebinthifolius* is a tree that is almost 8 m in height, and the diameter of the trunk can reach up to 60 cm. The leaves are composite by leaflets measuring 3 to 5 cm, and the plant has small flowers in a pyramidal structure and red fruits [57].	More frequently seen along the Brazilian coast from the north to the south and found in other regions such as Mato Grosso do Sul and Minas Gerais. It probably covers most of South America and was largely introduced in other countries, including the United States, as ornamental plants [57].

*Jacaranda decurrens* subsp. *symmetrifoliolata* Farias & Proenca	Bignoniaceae	The leaf extract (he) is described to have antiobesity, hypocholesterolemic, and hypolipidaemic [58] activities and the roots (he) has anti-inflammatory [59] activity.	The species measures 50-150 cm. Its leaf is biped, with leaflets elliptical to oblong, and its fruit is an oblong-obovate capsule, extremely woody, brown, and glabrous with a nonwavy margin in the dehiscence [60].	This is an endemic species of the southern State of Mato Grosso [60].

*Guazuma ulmifolia* Lam.	Malvaceae	The stem bark and leaf extracts (w) have antidiabetic potential [61]. The stem bark extract (a) has hypotensive, vasorelaxant [62], and gastroprotective [63, 64] effects.	The leaves *of G. ulmifolia* display as an ovoid structure; the flowers have long filiform appendages; and the black fruits have a capsular form of two to three centimeters [65].	*G. ulmifolia* is a tree that is distributed from Mexico to Brazil [65].

Legend. Solvent: e: ethanol; h: hexane; w: water; a: acetate; m: methanol; e : w: ethanol : water; h : e: hydro : ethanol; c : m: chloroform : methanol. Fraction: aw-F: acetate water; etacF: ethyl acetate fraction; meF: methanol fraction.

**Table 2:** Cytotoxic potential and compounds identified from extracts of Cerrado plants.

Plant species	Parts used	Model	Cytotoxic features	Compounds identified	Ref.
*S. adstringens*	Stem bark	B16F10-Nex2	Mitochondrial depolarization, caspase-3 activation, and ROS production	Gallic acid, procyanidins, and catechins	Baldivia et al. [16]
	Stem bark	HeLa, SiHa, and C33A	Intense oxidative stress, mitochondrial damage, increased Bax/BCL-2 ratio, and increased caspase-9 and caspase-3 expression	Proanthocyanidin polymer-rich fraction	Kaplum et al. [146]
		MCF-7 and MDA-MB-435	Increased Bax/BCL-2 ratio and increased caspase-9, active caspase-3, caspase-8, LC-3, and beclin-1 expression	Gallic acid, procyanidins, and catechins	Sabino et al. [147]

*C. adamantium*	Leaves	K562 cells	Caspase-3 and caspase-9 activation, cell cycle arrest at the S and G2 phases, and calcium influx	O-Pentoside and O-deoxyhexoside myricetin, quercetin O-pentoside, and myricetin-O-(O-galloyl)-pentoside	Campos et al. [17]
	Roots			O-Pentoside, O-methyl ellagic acid, O-hexoside, O-deoxyhexoside, O-methyl ellagic acid, and gallic acid	
	Leaves	PC-3	Inhibited prostate cancer cell proliferation, DNA fragmentation, and decreased NFkB1 expression	Chalcone cardamonin	Pascoal et al. [144]
		MCF-7, HeLa, and M059J	Inhibited cancer cell proliferation	*β*-Myrcene, spathulenol, germacrene-B, *β*-caryophyllene oxide, *β*-caryophyllene, *α*-pinene, viridiflorol, limonene, and (Z,E)-farnesol (6.51%)	Alves et al. [145]

*S. velutina*	Leaves	Jurkat/K562 cells	Caspase-3 activation, mitochondrial depolarization, cell cycle arrest at the S and G2 phases, and calcium influx	Epigallocatechin, epicatechin, rutin, kaempferol glycosides, and dimeric and trimeric proanthocyanidins	Campos et al. [18]
	Roots	B16F10nex2 cells and mouse C57b1/6	Increased intracellular ROS levels, induced mitochondrial membrane potential dysfunction, activated caspase-3, and impaired pulmonary metastasis *in vitro*	Flavonoid derivatives of catechin and piceatannol (active metabolite of resveratrol) groups and dimeric tetrahydroanthracene derivatives	Castro et al. [142]

*J. decurrens*	Leaves	K562 cells	Mitochondrial depolarization, Caspase-3 activation, necrosis and late apoptosis	Phenolic compounds and flavonoids	Casagrande et al. [19]

*H. speciosa*	Leaves	Kasumi-1 cells	Necroptosis and cathepsin release	Bornesitol, quinic acid, chlorogenic acid, and flavonoids derived from kaempferol and rutin	Dos Santos et al. [46]

*G. ulmifolia*	Stem bark	K562 cells and mouse C57b1/6	Protected against the doxorubicin-induced cardiotoxicity and reduced oxidative haemolysis *in vitro*	Citric and quinic acids	Dos Santos et al. [22]
	Leaves			O-Pentosyl and di-O-deoxyhesosyl-hesosyl quercetin, O-deoxyhexosyl hexosyl luteolin, and di-O-deoxyhexosyl hexosyl kaempferol	

*S. terebinthifolius*	Leaves	K562 cells and mouse C57b1/6	Protected against doxorubicin-induced cardiotoxicity and reduced oxidative haemolysis *in vitro*	Phenolic compounds, flavonoid, tannin, and ascorbic acid [21] and *α*-pinene, limonene, carene, and phellandrene [159]	Rocha et al. [21] and Carneiro et al. [159]
